# Phylostratigraphic tracking of cancer genes suggests a link to the emergence of multicellularity in metazoa

**DOI:** 10.1186/1741-7007-8-66

**Published:** 2010-05-21

**Authors:** Tomislav Domazet-Lošo, Diethard Tautz

**Affiliations:** 1 Max-Planck Institut für Evolutionsbiologie, August-Thienemannstrasse 2, 24306 Plön, Germany; 2 Laboratory of Evolutionary Genetics, Division of Molecular Biology, Ruđer Bošković Institute, Bijenička cesta 54, PP 180, 10002 Zagreb, Croatia

## Abstract

**Background:**

Phylostratigraphy is a method used to correlate the evolutionary origin of founder genes (that is, functional founder protein domains) of gene families with particular macroevolutionary transitions. It is based on a model of genome evolution that suggests that the origin of complex phenotypic innovations will be accompanied by the emergence of such founder genes, the descendants of which can still be traced in extant organisms. The origin of multicellularity can be considered to be a macroevolutionary transition, for which new gene functions would have been required. Cancer should be tightly connected to multicellular life since it can be viewed as a malfunction of interaction between cells in a multicellular organism. A phylostratigraphic tracking of the origin of cancer genes should, therefore, also provide insights into the origin of multicellularity.

**Results:**

We find two strong peaks of the emergence of cancer related protein domains, one at the time of the origin of the first cell and the other around the time of the evolution of the multicellular metazoan organisms. These peaks correlate with two major classes of cancer genes, the 'caretakers', which are involved in general functions that support genome stability and the 'gatekeepers', which are involved in cellular signalling and growth processes. Interestingly, this phylogenetic succession mirrors the ontogenetic succession of tumour progression, where mutations in caretakers are thought to precede mutations in gatekeepers.

**Conclusions:**

A link between multicellularity and formation of cancer has often been predicted. However, this has not so far been explicitly tested. Although we find that a significant number of protein domains involved in cancer predate the origin of multicellularity, the second peak of cancer protein domain emergence is, indeed, connected to a phylogenetic level where multicellular animals have emerged. The fact that we can find a strong and consistent signal for this second peak in the phylostratigraphic map implies that a complex multi-level selection process has driven the transition to multicellularity.

## Background

Genomic phylostratigraphy is an analysis method based on a model of punctuated evolution of protein families, which assumes that protein families are initiated by founder genes in a scattered manner throughout evolutionary time [1,2]. Founder genes in this sense are genes that represent evolutionary novelties in protein sequence space [1,3,4] - that is, are not simply duplications of existing genes or genes with re-shuffled functional domains. Rather, they represent new functional proteins or protein domains that were previously not present in the genome, at least not in the new functional form. Once such a new functional domain has emerged, it would be expected that it would retain its primary protein sequence to an extent where it would still be traceable by sequence similarity searches [3].

Major evolutionary innovations are expected to be accompanied and, at least partly, caused by the emergence of founder genes. Indeed, we were, for example, able to track the macroevolutionary origin of the nervous system and the germ layers based on expression data and the phylogenetic classification of *Drosophila *genes [1]. Evidently, the genetic architecture of any complex phenotype will also include co-opted genes that have arisen before or after the respective phenotypic innovation occurred. However, they are expected to be co-opted at lower rates and different times and, thus, contribute to the phylostratigraphic signal to a lesser extent. This allows the origin of a phenotypic innovation to be discerned on the phylostratigraphic maps [1] (see Methods for a more detailed description of the procedure).

Multicellularity is a complex phenotype and considered to be one of the major evolutionary transitions [5]. It seems that multicellularity evolved independently dozens of times in different lineages [5,6], whereby the multicellular lineage leading to animals (metazoa) is thought to have emerged from a unicellular Choanoflagellate-like ancestor [7].

Cancer is thought to be a probabilistic event determined by a series of mutations occurring in cancer-associated genes and it seems that a few thousand genes could contribute to tumour development [8,9]. However, mechanistically these genes do not all contribute in the same way to cancer progression. On a broad scale, two major groups were proposed: caretakers and gatekeepers [10,11]. Mutations in caretakers promote tumour progression in an indirect way by increasing mutation rates and genome instability, which increases the chances that mutations will hit some genes within the gatekeepers. Mutations in gatekeepers promote tumour progression directly by changing cell differentiation, growth and death rates. Gatekeepers can be further classified into oncogenes and tumour suppressor genes. It is often assumed that cancer in animals is a legacy of the evolution of a multicellular life style [12] but it is fairly unexplored whether tumours could also be found in early branching metazoans [13,14]. Moreover, a possible direct link between the macroevolutionary origin of multicellularity and cancer is not necessarily predicted, since the genes involved in causing cancer could have emerged independently at any time during evolution.

We have tested here whether the emergence of founder genes related to cancer has a correspondence to the evolutionary origin of multicellularity. We find a surprisingly clear signal of gene emergence that corresponds well with the major classification of cancer genes. This gives both credence to the classification and the notion that cancer is an ancient phenomenon with a direct correspondence to basic cell function and the interaction of cells.

## Results

### Phylostratigraphy of cancer genes

**Figure 1:**
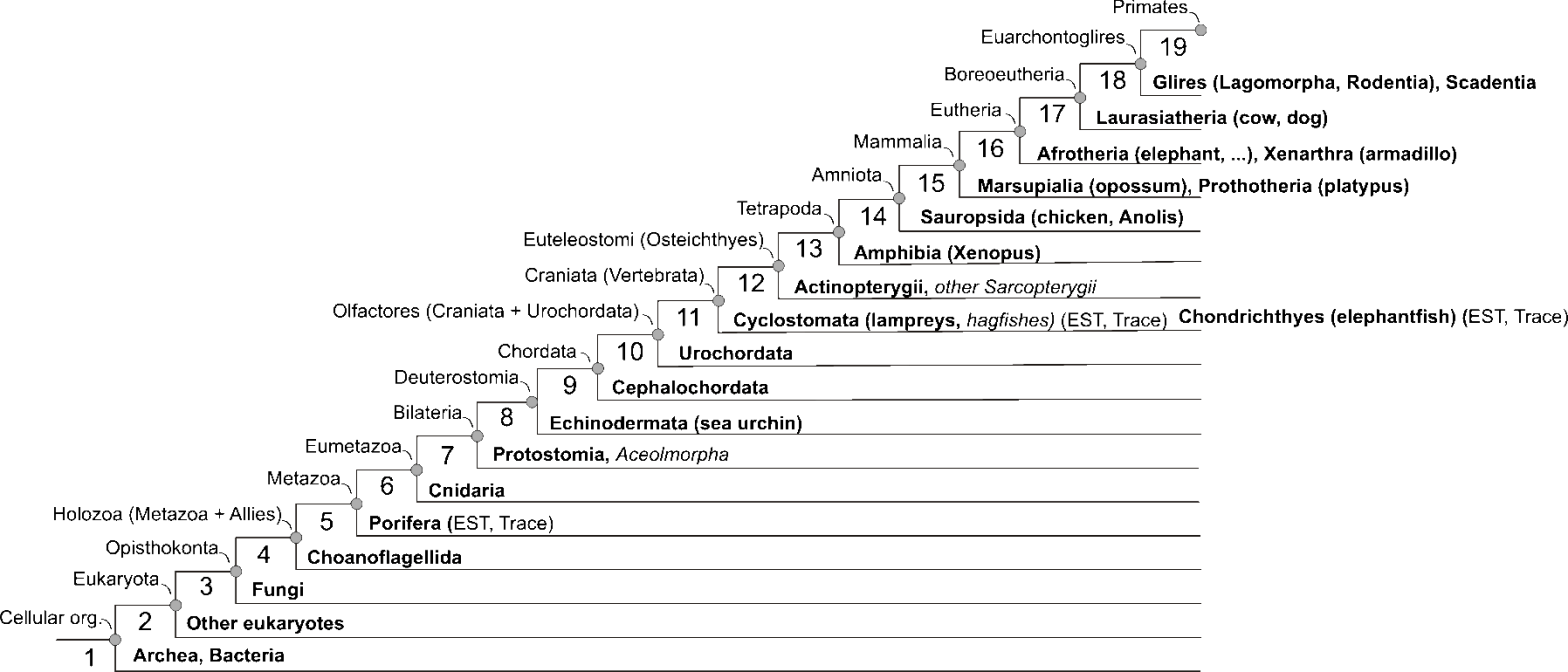
**Phylogeny used in the search for the evolutionary origin of human genes**. Taxa represented in the databases with complete genomes or a substantial amount of TRACE and expressed sequence tag data are in bold. Taxa in italics are represented in the databases only with small numbers of highly conserved genes: their exclusion from the analysis does not influence the results. The phylogeny is based on the results of the most recent phylogenomic analyses [15-19].

Based on our previously described phylostratigraphic procedure [1,2], we generated a database of genome sequences divided into 19 phylostrata corresponding to the evolutionary relationships of the major taxa supported by phylogenomic analyses (Figure 1) [15-19]. This was then used to trace the evolutionary origin of cancer genes identified in humans. If the origin of human cancer genes carries a phylostratigraphic signal related to the macroevoutionary origin of cancer, this should be apparent on the phylostratigraphic map as a distinct and significant overrepresentation of such genes in a particular evolutionary period [1,2].

**Figure 2:**
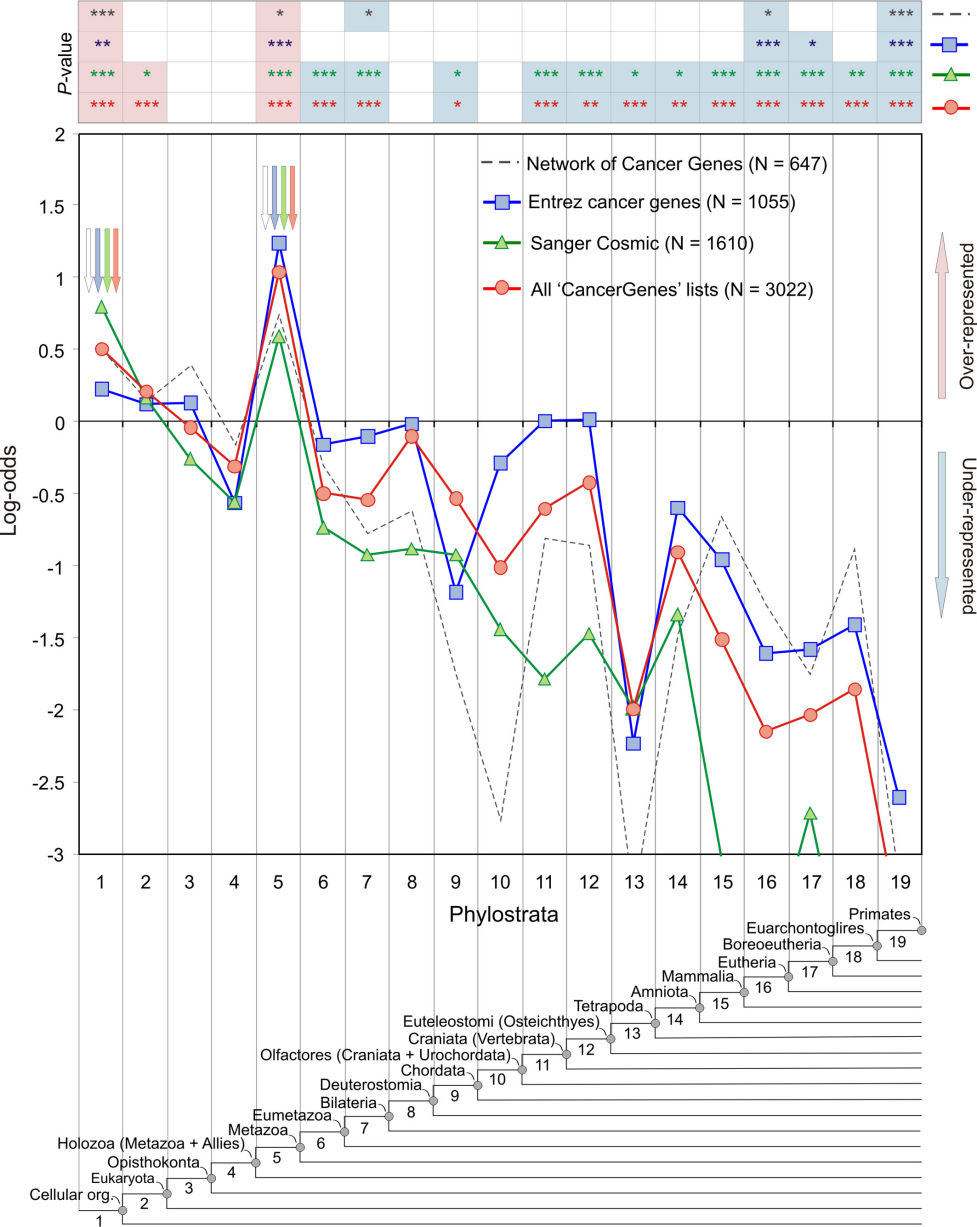
**Statistical analysis of the cancer datasets on the phylostratigraphic map**. Phylostratigraphic representation of log-odds statistics of human cancer genes, based on four different compilations (see inset on top right). Arrows designate the strongest significant over-representations. Statistical significance of the deviations were tested by a two-tailed hypergeometric test corrected for multiple comparison by a false discovery rate at 0.05 level (* *P *< 0.05; ** *P *< 0.01; *** *P *< 0.001). Significant over-representations and under-representations are shaded in red and blue, respectively.

A substantial number of genes associated with cancer have been reported in public databases and these databases vary in scope, level of curation and inherent biases [20-23]. Still, surprisingly similar phylostratigraphic patterns were obtained for all of them. In order to illustrate this robustness, we show the phylostratigraphic profiles derived from four compilations of cancer associated genes that represent a spectrum of stringency levels for inclusion of genes (Figure 2). The first dataset contains genes found to be mutated in human tumours (Sanger Cosmic) and the second one includes human genes with cancer related annotation in the National Center of Biotechnology Information databases (Entrez section in CancerGenes). The third most inclusive dataset includes the previous two datasets plus genes involved in cancer biochemical pathways and cancer associated biochemical functions (CancerGenes). The fourth one represents an intensively curated dataset that also includes system properties of cancer genes (Network of Cancer Genes).

The phylostratigraphic profiles of all four are highly congruent and show two strong over-representation peaks - one at the origin of the first cells (phylostratum 1 = ps1) and the other at the origin of the metazoa (ps5). A significant over-representation is also seen at the level of the emergence of eukaryota (ps2) but it is not as strong as that at ps1. No further over-representation occurs after ps5. On the contrary, the origination of founder genes related to cancer is significantly lowered at the emergence of the eumetazoa and bilateria (ps 6 and 7) and at all levels beyond the emergence of vertebrates (ps11 onwards) (Figure 2).

### Origin of metazoa and cancer

The above result suggests that the two fundamental events for the emergence of cancer-related genes were the origin of the first cell and the origin of the stable form of multicellularity in metazoans. Although multiple transitions to multicellularity may have occurred before the emergence of the metazoa [fungi (ps3) and Chanoflagellata (ps4)], it seems likely that these were independent and reversible events [5-7]. The metazoan form of multicellularity, on the other hand, has apparently been stable throughout evolution and, thus, may have been a key innovation, including many adaptive changes that required the recruitment of new genes [5,6]. This would explain the strong peak of cancer gene emergence seen at ps5.

An unexpected peak with respect to cancer genes is the one in ps1, the origin of the first cells. Genes that have emerged at this time would not usually be considered to have a role in regulating multicellularity. However, this peak could make sense in the light of the classification of cancer genes into caretakers and gatekeepers [10,11]. Caretakers could have evolved earlier, since their genome stability functions are of general importance for a cell, probably independent whether or not it is part of a multicellular organism. Gatekeepers, on the other hand, fulfil functions that are related to influencing cooperation among cells (oncogenes) or to prevent the expansion of cheater cells (tumour suppressor genes). One could predict that both of these gatekeeper functions would be necessary for stable multicellularity [24-26] and should, therefore, have predominantly arisen at the time of emergence of metazoa.

**Figure 3:**
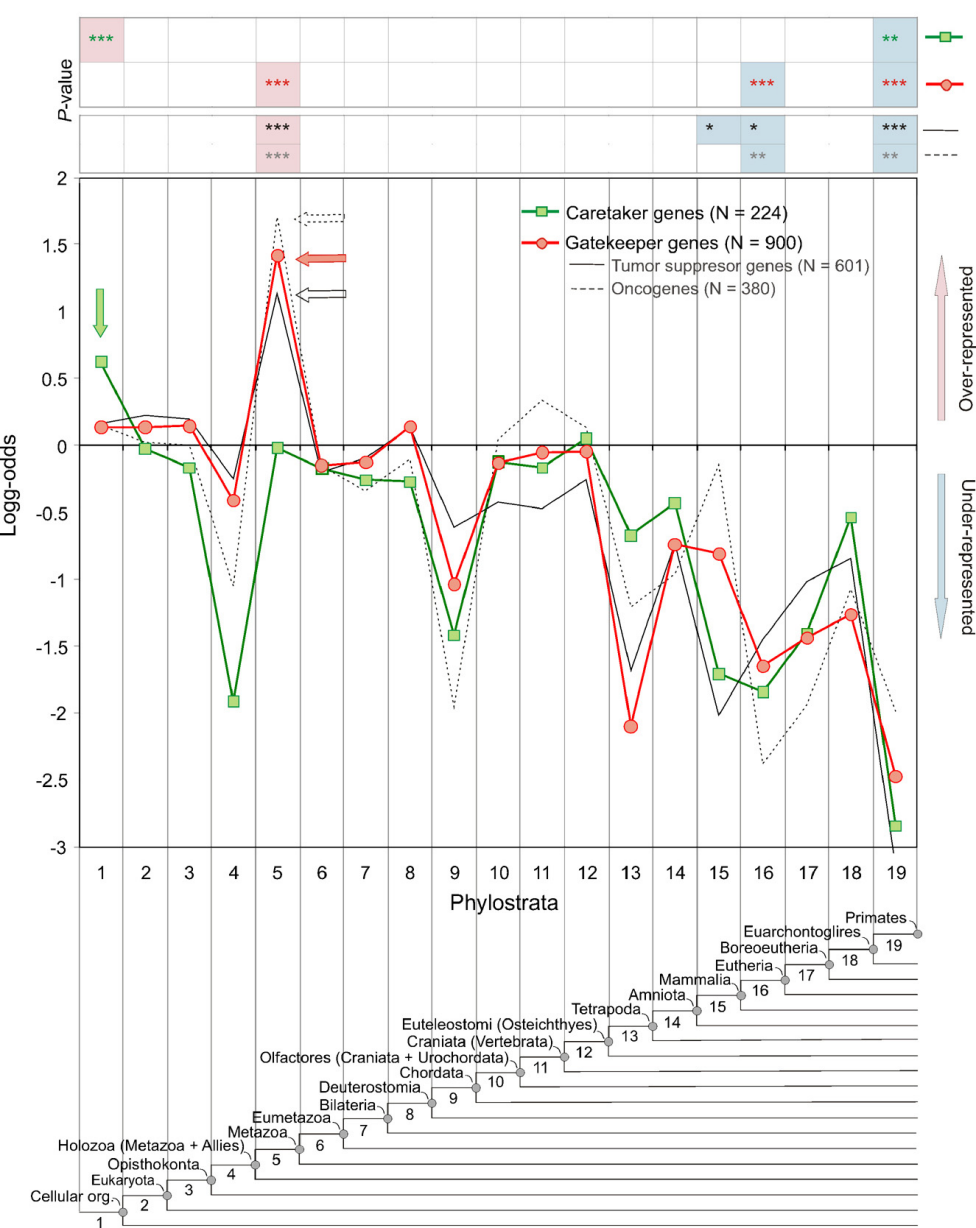
**Statistical analysis of caretakers and gatekeepers on the phylostratigraphic map**. Phylostratigraphic representation of log-odds statistics of human caretaker and gatekeeper cancer genes following the annotations in Entrez are shown. Arrows designate the strongest significant over-representation. Squares denote the caretaker dataset (green line, *N *= 224) and circles denote the gatekeeper dataset (red line, *N *= 900). The gatekeeper dataset is further subdivided into tumour suppressor genes (solid black line, *N *= 601) and oncogenes (dashed line, *N *= 380). Statistical significances of the deviations were tested as described in the legend of Figure 2.

In order to test these predictions, we analysed the different categories of cancer genes separately. We find that the origin of gatekeeper functionality does, indeed, correspond to the origin of metazoa (ps5), with no other significant peak (Figure 3). The further subdivision of this class into oncogenes and tumour-suppressor genes confirms this picture (Figure 3). Both display a peak in their emergence only at ps5, in line with the notion that they are both required together. Caretaker functionality, on the other hand, is predominantly associated with the emergence of the first cellular organisms (ps1) with no further significant peak (Figure 3). This suggests that the cellular machinery that secures the stability of the genome in the context of cancer development was, indeed, in place a long time before multicellularity in animals and gatekeeper functionality evolved.

## Discussion

The systematic compilation of genes involved in cancer has used very different criteria for inclusion of genes, which are reflected in an almost fivefold difference in gene numbers between the two most extreme sets. In spite of these differences, we note that all currently available systematic lists provide very congruent patterns in the phylostratigraphic analysis. This suggests that there is an underlying robust pattern, both in our analysis, as well as in the compilations. This was also found in other recent studies to uncover common properties of cancer genes [27,28]. Still, there are several inherent assumptions in the phylostratigraphic approach that require consideration.

### Technical considerations

One assumption concerns the validity of the phylogeny. For the results we presented here, the early cladogenesis around the Metazoa is particularly critical. In our analysis we took the classical view, where Porifera are a basal metazoan clade, whereas Cnidaria and Bilateria are branching off later. However, some recent studies found a limited support for an alternative hypothesis, namely that Porifera together with Cnidaria are the earliest branching clade, while Bilateria is a sister group to these diploblastic animals [29,30]. Although general phylogenomic analyses do not support this view [15,16,19], we tested also this alternative hypothesis. We find that our phylostratigraphic pattern is not much affected by this different topology: gatekeeper genes are still significantly over-represented at ps5 and not in other phylostrata (data not shown).

Since a comprehensive cancer gene catalogue is currently only available for humans, our phylogeny is necessarily focused towards humans and presented in a way that tries to depict the major transitions from unicellularity to humans. Once similar comprehensive cancer gene datasets become available for other species - for example, a plant species - one would evidently build a different phylogeny to capture the major transitions towards this species. Another constraint is the availability of fully sequenced genomes. As long as a phylostratum is covered by a single genome only, there is always the chance that this particular species has lost a gene, which would otherwise be present in its lineage. The classification of the respective gene would therefore fall into the next phylostratum and it would seem to be younger than it is. In order to tackle this problem, at least partly, we have also included expressed sequence tag (EST) databases of additional species from a given phlyostratum, if genomic coverage was low.

Another major concern is whether the BLASTP analysis does, indeed, capture the most remote homologues. Although we used a rather permissive cut-off, there are more refined iterative programmes, such as PSI-BLAST, which use profile information from aligned sequences to find remote homologues. However, the increased sensitivity results at the same time in a higher false positive rate [31]. However, there is also a conceptual reason why we prefer BLASTP. We have previously argued that novel gene functions should be associated with novel lineage-specific processes and that the proteins involved in them should have gone through a rapid phase of evolutionary optimization, even if they were initially created by a duplication [1,3]. Algorithms that are designed to detect distant relationship between proteins, like PSI-BLAST, would be the choice if one would be interested in the ancestral gene duplication that preceded the formation of a founder domain. However, we are interested in the event of founder domain formation *per se*, which we expect to be characterized by a shift in sequence space, where a substantial proportion of amino acid sites has changed. Once a founder domain has emerged through a duplication event and fast divergence [3] or through *de novo *formation [32-34] one would expect normal clock-like divergence, with a constraint on the functional domain and this is indeed the evolutionary pattern that is readily detected by BLASTP searches [35]. However, there is also an inherent weakness of our approach. If a novel gene function is created by the recombination of different functional domains, we would place the origin of this gene into the phylostratum where the domains have originated. Thus, these genes would not be correctly placed in the context of the biological process within which they have emerged. However, this effect, as well the genomic under-representation effect discussed above, is not expected to create a particular bias towards certain phylostrata and would thus only contribute noise to the analysis.

A further source of uncertainty is the correct classification of genes into a given process. In our case, we have to expect that there is an overlap within the classification of gatekeepers and caretakers. For example, for one of the best studied cancer genes, p53, it is becoming clear that it has a multitude of functions [36], which makes a simple classification into one of the above categories difficult. It is to be expected that this will also be the case for other genes, once their full functional spectrum is understood. However, the over-representation analysis at the functional level that we applied here allows multiple annotations for a single gene and, therefore, inherently overcomes this type of problems.

Given that all these factors should blur our analysis, it is even more surprising that we still find significant associations between gene emergence and biological processes, such as evolution of multicellularity. This suggests that there is indeed a strong underlying signal and that the inevitable noise in the analysis does not override it.

### Emergence of multicellularity

From the theoretical point of view, transition to multicellularity represents an increase in hierarchical complexity of an organism, where cells become cooperatively organized in collectives [24-26]. This transition inherently faces cross level conflicts stemming from dependencies between individual cells and collective fitness. At final stages of the transition process, where collectives are fully emerged entities with their own life cycles, single cell and collective fitness are largely decoupled. However, to reach this final stage of transition to multicellularity both cooperation promoting and conflict reducing adaptations are needed [24-26]. In this context, it has been speculated that the emergence of multicellular life should also have brought about genes that control cheater cells [24]. The function of gatekeepers could be directly associated with the control of cheater cells and it is, therefore, of special interest that we can indeed trace the peak of their appearance to the emergence of metazoan life. In fact, although it seems intuitively clear that cancer genes and multicellularity should somehow be connected, this intuition makes no specific prediction at which time point during the evolution of multicellular lineages one would have expected cancer genes to emerge. Our analysis shows that there is indeed only a subset of cancer genes that are directly connected to the emergence of multicellularity. However, the functional categories associated with this subset imply that there must have been a multi-level selection process [24-26] that was active at the basis of the metazoan lineages, involving the evolution of a multitude of new genetic processes and gene functions connected to the interactions between the cells.

### Macroevolutionary considerations

**Figure 4:**
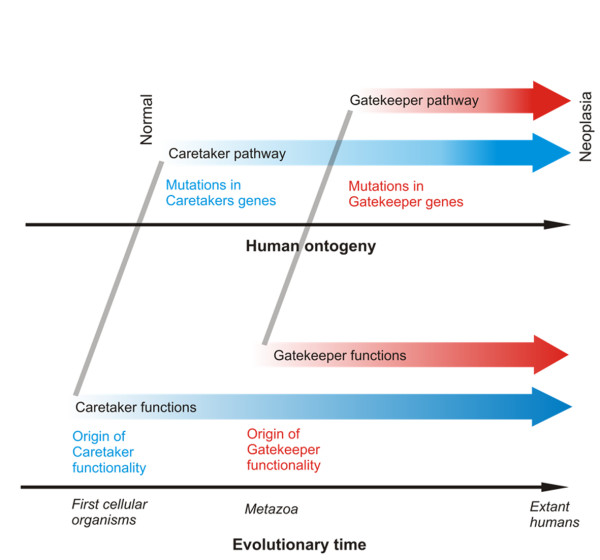
**Colinearity between evolutionary age of cancer genes and their role in tumour progression**. Parallels between the macroevolutionary origin of the global pathways leading to neoplasia and the developmental timing of mutations in these pathways are shown. The upper part of the figure is adapted from reference [11].

It has been noted that there is a developmental timeline of mutations in caretaker and gatekeeper pathways during tumour progression [37-39]. Caretaker mutations tend to precede gatekeeper mutations and may in fact facilitate these, although this notion has also been disputed [38]. In the context of the macroevolutionary origin of these functionalities, an intriguing parallel between this assumed cellular progression and the phylogeny of these processes is apparent (Figure 4), supporting the notion that there is indeed a successive role for these groups of genes in carcinogenesis.

We have previously performed a similar phylostratigraphic study on the emergence of genes involved in genetic diseases [2], with the major finding being that they have arisen very early in evolution, with peaks in ps1 and ps 4/5. Almost no new disease associated genes emerged after the evolution of mammals (ps15). This is a similar finding to that reported here, although the overlap between the disease gene list and the cancer gene lists is only 10%-15%. Hence, cancer genes and disease genes constitute different classes of genes but have similar macroevolutionary histories.

It has been proposed that cancer can be seen as an evolutionary and ecological process within an individual [40] and typical methods of evolutionary analysis have been applied in order to understand its progression [12,41,42]. Our analysis provides a link to the evolution of the gene functions that were required to assemble the first metazoan organism and, thus, adds a macroevolutionary perspective for the emergence of evolutionary dynamic processes controlling complex life forms.

## Conclusions

Phylostratigraphic analysis is a potentially powerful tool to better understand the genetic basis of evolutionary transitions, although it focuses only on one aspect of novel gene emergence, namely the appearance of founder protein domains. It will further increase its utility when more fully sequenced genomes are available throughout the tree of life. However, the above described pattern for the emergence of founder genes involved in cancer is already now very robust and confirms the ancient origin of gene functions involved in cancer.

## Methods

### The phylostratigraphic procedure

Phylostratigraphic analysis was basically done according to the procedures described in our previous studies [1,2]. In short, the procedure and its theoretical underpinnings are as follows. The procedure is essentially done in two steps. The first step involves the creation of a consensus phylogeny, where each node is represented by one or more fully sequenced genomes (supplemented with EST data, where necessary). The origin of all genes from an extant genome which is the focus of the analysis (in our case humans) are then mapped to a particular node in this phylogeny (called phylostratum), based on BLASTP analysis. This creates a null distribution of founder protein domain emergence. In the second step, one recovers a distribution of genes that are connected to a certain phenotype (in our case cancer) and uses a statistical test to assess for every phylostratum the way in which this distribution deviates from the null distribution.

Although this seems like a straight forward procedure, it requires various considerations and assumptions to be workable, in particular with respect to the question of how to assign the true evolutionary origin of a given gene. In principle, the mapping of genes onto the phylogeny could be performed in different ways depending on which criteria of evolutionary relatedness are used to define homology groups. For example, very distantly related proteins could be assigned to a protein family based on the similarity of their structural properties even if their primary sequences diverged below similarity detection level. On the other side of the spectrum of possibilities would be the quest to identify truly orthologous proteins with matching domain architectures. The criteria for grouping will largely depend on the evolutionary question one is interested in. In the phylostratigraphic approach, where the statistical signature of macroevolutionary adaptations is the focus, we are applying a procedure somewhere in between these extremes. We group proteins according to any detectable homology between sequences - that is, according to the uniqueness of their sequence in the protein sequence space. This threshold is in protein science sometimes called the 'twilight zone'' of sequence similarity [43,44]. It implies, for example, that for a multi-domain protein its origin is mapped to the age of its oldest domain and we are interested in the emergence of this very first function.

Our focus on such founder protein domains is a central tenet of the model of punctuated evolution of protein families [1]. The underpinning idea of this model is that new genes with completely novel sequences are continuously added in a genome throughout evolutionary time [45,46]. This could occur due to gene duplication with a subsequent fast divergence of one of the duplicated copies in the context of a new adaptation [3,4], or even due to the conversion of intergenic sequences into functional ones [32-34]. Once such a new gene with a novel sequence (or domain) is stably incorporated into a functional circuit it is considered to be a founder gene and can eventually contribute to the formation of paralogs or rearrangements with other genes. As a founder acts as a seed for the later proliferation of similar descendant genes, any future protein that contains it, or part of it, belongs to its family. Thus, in the phylostratigraphic approach, we intentionally do not distinguish between orthologs and paralogs, but are specifically interested in the emergence of the founder gene itself, since it is to be expected that its appearance is tightly associated with the appearance of an evolutionary innovation. An additional support for this view comes from the recent work that suggests that there is generally no clear cut between paralogs and orthologs in their divergence patterns and functional change [47].

### Phylostratigraphic analysis

In order to be on the more conservative side of the human genome annotation errors, we retrieved from public databases the same compilation of human protein sequences (20,259 unique proteins) that were used in the recent cancer genome sequencing projects [48]. The BLASTP algorithm (0.001 E-value cut-off) was used to compare human proteins against the NCBI NR database (see [1,2] for the choice of BLASTP and the cut-off value). This database represents the most exhaustive set of known proteins across all organisms and is, therefore, the most suitable for phylostratigraphic analysis. Before the sequence similarity search was done, the NR database was cleaned up with respect to sequences with uncertain taxonomic status (for example, those annotated as 'incerteae sedis', 'environmental samples' or 'unclassified') or where the taxonomy ID is not included in the cellular organisms section of the NCBI taxonomy database. Additionally, we removed from the database sequences of metazoan taxa with currently unreliable phylogenetic position (Mesozoa, Myxozoa, Chaetognatha and Placozoa). After this clean up procedure we filled up the NR database with complete genomes which were absent in the database but were otherwise publically available [2]. The curated NR database finally contained 4,749,457 protein sequences.

In addition, the TBLASTN searches (10^-15 ^E-value cut-off) were done against substantial trace and EST archives of Porifera, Cyclostomata and Chondrichtyes (phylostrata 6 and 11, Figure 1) as complete annotated genomes are still lacking for these internodes. The higher threshold for the trace and EST archives was necessary because of the different data structure.

Using the obtained BLAST output and the MS SQL database management system in a series of queries we mapped human genes according to the evolutionary origin of their founder genes on the currently best supported phylogeny (Figure 1) [15-19]. Taken together, our choice of internodes depended on the availability of complete annotated genomes, reliability of phylogenetic relationships and on the importance of evolutionary transitions.

### Retrieval of cancer associated genes

We retrieved cancer associated genes from several public resources [20-23]. The full list of genes and datasets used in the analysis is listed in Additional File 1: Table S1. We performed a series of overrepresentation analyses for various combinations of these datasets, in a way that the frequency of cancer associated genes in every phylostratum was compared to the frequency of cancer associated genes in the complete genome (expected frequency) [1,2]. Obtained deviations are shown by calculating log-odds ratios and their significance were tested by two-tailed hypergeometric tests [49] corrected for multiple comparisons via a false discovery rate at the 0.05 level [50]. The caretaker and gatekeeper classifications were taken from the Entrez section in the CancerGenes database [20] as this was currently the only large scale annotated dataset that allowed this type of grouping in a straightforward way. Note that the distinction between caretakers and gatekeepers is not definitive; some genes were listed in both of these categories as they act as both caretakers and gatekeepers. However, all of this information is taken into account and does not pose a problem for the statistical treatment in the overrepresentation analysis. For the analysis of caretakers and gatekeepers we used a more stringent subset of Entrez CancerGenes by taking into account only those that have definitive evidence that they are mutated in human tumours (Sanger Cosmic list [22]). However, similar results are also obtained when the other databases were used (data not shown).

## Abbreviations

EST: expressed sequence tag; NCBI: National Center for Biotechnology Information.

## Authors' contributions

TDL initiated the study and carried out all the bioinformatic analysis. DT contributed to the data interpretation and further development of the study. TDL and DT wrote the manuscript.

## Supplementary Material

Additional file 1 **Table S1**. The file lists all the human genes that were considered in this analysis and associates them with their respective phylostratum. It further contains the lists of genes from all four databases used and the assignments to categories. Click here for file
